# Network Controllability-Based Prioritization of Candidates for SARS-CoV-2 Drug Repositioning

**DOI:** 10.3390/v12101087

**Published:** 2020-09-26

**Authors:** Emily E. Ackerman, Jason E. Shoemaker

**Affiliations:** 1 Department of Chemical and Petroleum Engineering, University of Pittsburgh, Pittsburgh, PA 15260, USA; eea16@pitt.edu; 2 The McGowan Institute for Regenerative Medicine (MIRM), University of Pittsburgh, Pittsburgh, PA 15260, USA; 3 Department of Computational and Systems Biology, School of Medicine, University of Pittsburgh, Pittsburgh, PA 15260, USA

**Keywords:** SARS-CoV-2, drug repositioning, COVID-19, network biology, virus-host interactions

## Abstract

In a short time, the COVID-19 pandemic has left the world with over 25 million cases and staggering death tolls that are still rising. Treatments for SARS-CoV-2 infection are desperately needed as there are currently no approved drug therapies. With limited knowledge of viral mechanisms, a network controllability method of prioritizing existing drugs for repurposing efforts is optimal for quickly moving through the drug approval pipeline using limited, available, virus-specific data. Based on network topology and controllability, 16 proteins involved in translation, cellular transport, cellular stress, and host immune response are predicted as regulators of the SARS-CoV-2 infected cell. Of the 16, eight are prioritized as possible drug targets where two, PVR and SCARB1, are previously unexplored. Known compounds targeting these genes are suggested for viral inhibition study. Prioritized proteins in agreement with previous analysis and viral inhibition studies verify the ability of network controllability to predict biologically relevant candidates.

## 1. Introduction

As COVID-19 spreads worldwide with 25 million cases and 800,000 deaths occurring between January and August 2020 [1], there is an urgent need for novel treatment options. There are currently no known pharmaceutical treatments for SARS-CoV-2 infection. One strategy to accelerate the identification of possible leads is to reposition drugs with known targets and mechanisms that may have been through parts of the FDA approval process [2]. Avoiding this development pipeline known for its low success rate [3] advantageously saves invaluable time and monetary cost. While this is ultimately the fastest way to get treatments to patients in need, the most efficient way to discover drugs with the potential for repurposing is unclear.

In the short time since the beginning of the pandemic, many attempts to predict candidate drugs for repositioning have been made. Given the novel nature of the virus, methods of target prediction have been forced to utilize the limited data that is available or creatively repurpose data from related coronaviruses. In vitro screenings of chemical libraries have been used to identify inhibitors of SARS-CoV-2 replication [4,5] and cellular toxicity [6]. Screenings of experimentally verified SARS-CoV-2 interacting host proteins [7] have elucidated key infection mechanisms which, when compared to drug databases, have predicted a range of possible targets for repurposing. Network analyses using protein interaction data from up to 13 related human coronaviruses [8,9] combined with in vitro screenings have identified additional sets of cellular pathways to consider for drug repurposing. Topology of protein interactions and drug–gene interactions combined with differential expression and pathway analysis has been used to identify possible mechanisms of action for SARS-CoV-2 infection [10]. With each method integrating varying levels of biological detail, overlap between studies is optimal for ensuring infection-specific relevance and effectiveness.

Here, the existing methods for identifying influenza A virus drug targets [11] are applied to SARS-CoV-2–human host protein interaction data to predict and prioritize candidate targets for drug repurposing. Two methods of network controllability determine the identity of proteins acting as regulators of the infected cell marked by changes to the network’s behavior after the addition of virus–host protein interactions. Both methods use a maximum matching algorithm (e.g., Hopcroft-Karp) to identify the “driver nodes” of the network which must be manipulated for the system to be fully controlled (analogous to the non-zero elements of the state space B matrix of classic control systems engineering [12]). These nodes, specified by the paths which span the maximum amount of the network with no node sharing two edges, dictate the “easiest” way in which control can propagate through the network. Directing total system behavior is impossible without manipulating all driver nodes of the system at once. Of note, driver sets are typically not unique with the number of driver node sets scaling exponentially with the size of the network [13]. As a result, each driver node set (size $N_{D}$) can also be referred to as a minimum input set (MIS).

The analysis contains two methods of controllability. In robust controllability [14], each node of the network is removed, the driver set is re-calculated (size $N_{D}^{\prime }$), and the removed node is classified by its effect on the changes to the size of the driver set. Increasing the number of driver nodes ($N_{D}^{\prime }>N_{D}$) makes it more difficult to control the network (these nodes are classified as indispensable nodes) and decreasing the number of driver nodes ($N_{D}^{\prime }<N_{D}$) makes it easier to control the network (these nodes are classified as dispensable nodes). A removed node with no effect on the number of driver nodes ($N_{D}^{\prime }=N_{D}$) is classified as a neutral node. This method provides information concerning the structural robustness of the network and the effect of losing singular network components. A second method, global controllability [15], classifies each node by its membership to all possible MISs of the network. Critical nodes are included in all of the network’s possible MISs, intermittent nodes are only included in some of the possible MISs, and redundant nodes are not included in any of the possible MISs. Therefore, this method presents information about alternative methods of network control.

A comparison of the controllability of the human protein–protein interaction network (Host Interaction Network, HIN) and the human network with the addition of SARS-CoV-2–host protein interactions (Virus Integrated Network, VIN) can be used to identify proteins with unique post-infection roles in driving total cell behavior. Assuming the identified differences are representative of biological changes within the cell (such as changes to gene regulation), the protein predictions have potential as virus-specific drug targets. Here, 16 proteins are identified by topological, controllability, and biological relevance to viral infection. Of these proteins, eight are prioritized for drug repurposing efforts to treat SARS-CoV-2 infection based on previous druggability and relevance to functions such as translation, cellular transport, and the immune response.

## 2. Methods

### 2.1. Protein–Protein Interaction Network Construction

The human protein–protein interaction network was published by Vinayagam et al. [16]. The network was restricted to interactions with a confidence level greater than 0.7 based on the correlation between confidence scores and biological relevance as discussed by Yu and Finley [17]. This serves as the Host Interaction Network (HIN). Host proteins identified in the SARS-CoV-2–host interactions from Gordon et al. [7] were referenced against the host network. Interactions with host proteins that were present in the host network were added to form the virus–host network. This serves as the Virus Integrated Network (VIN). All analysis was completed in R 3.6.1 using the igraph package.

### 2.2. Robust Classification

Methods for robust controllability are sourced from Liu et al.’s work [14]. For any network with *n* total nodes, a subset, $N_{D}$, of driver nodes is found using a maximum matching algorithm such as Hopcroft–Karp on the bipartite representation of the total network [18]. This process is repeated iteratively after removing each node from the network ($N^{\prime }=N-1$) to identify a new maximum matching, *N_D_′*. Removed nodes are classified as indispensable ($N_{D}^{\prime }$ > $N_{D}$), neutral ($N_{D}^{\prime }$ = $N_{D}$), or dispensable ($N_{D}^{\prime }$ < $N_{D}$).

### 2.3. Global Classification

Calculations for Jia classification were adopted from Jia et al. [15]. For any network with *n* total nodes, a subset, $N_{D}$, of driver nodes is found using a maximum matching algorithm such as Hopcroft–Karp on the bipartite representation of the total network [18]. Control adjacent nodes of all $N_{D}$ are identified iteratively and used to create an input graph as described in Zhang et al. [19]. Nodes are classified as critical (in all minimum input sets), neutral (in some minimum input sets), or redundant (in no minimum input sets) based on the input graph.

## 3. Results

### 3.1. Topology of the Host Interaction Network and Virus Integrated Network

After construction (see Section 2), the Host Interaction Network (HIN) contains 6,281 proteins and 31,079 interactions. Median log_10_ degree and betweenness of the HIN is 0.699 and 2.945, respectively. In the construction of the Virus Integrated Network (VIN), 23 of the 27 SARS-CoV-2 proteins tested in Gordon et al. [7] are added to the network along with 152 interactions with 148 existing host proteins. Four SARS-CoV-2 proteins, spike, nsp11, ORF3b, and ORF7a, had no known interactions with host proteins of the HIN and were omitted from the analysis. In total, the VIN contains 6304 proteins and 31,231 interactions. Median log_10_ degree and betweenness of the VIN is 0.699 and 2.823, respectively. There is no statistical difference between the degree or betweenness of the HIN and VIN (Wilcoxon rank sum test p-values: 0.776 and 0.994, respectively). However, all host proteins have higher betweenness in the VIN compared to the HIN. While viral proteins only interact with 148 proteins, the topological effects are seen across the entire network.

**Figure 1:**
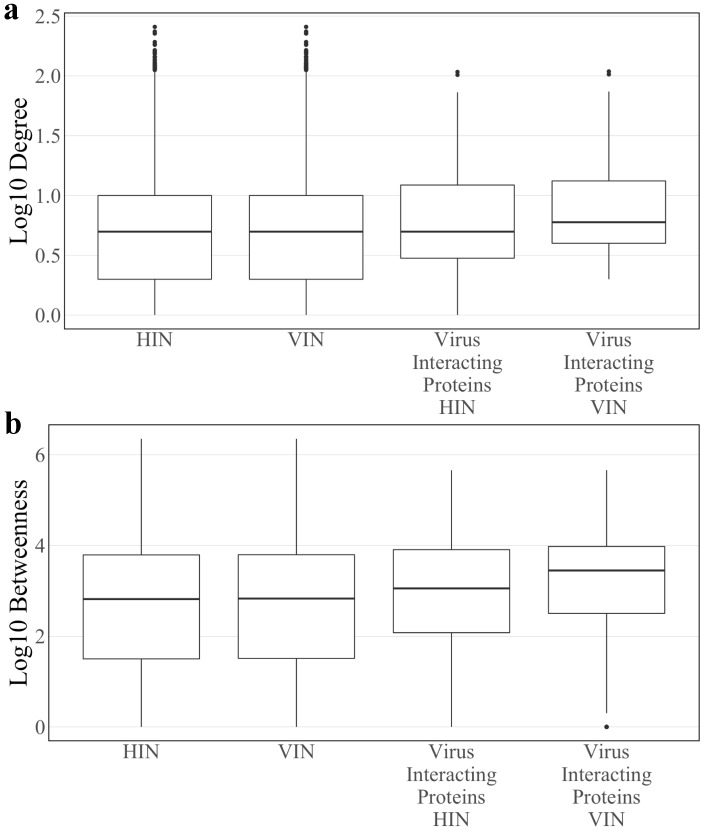
The log_10_ (**a**) degree and (**b**) betweenness distributions of the Host Interaction Network (HIN) and Virus Interaction Network (VIN) with the corresponding distributions for the subset of SARS-CoV-2 interacting host proteins.

Median log_10_ degree and betweenness of host proteins directly interacting with at least one SARS-CoV-2 protein, or “virus interacting proteins”, in the HIN are 0.699 and 3.053, respectively. The same values in the VIN are 0.778 and 3.449, respectively. There is a significant difference in mean degree and betweenness distributions of virus interacting proteins compared to the total protein population of the VIN (two sample *t*-test *p*-value: 3.02 × 10^−5^ and 1.801 × 10^−6^, respectively). All described degree and betweenness distributions are found in Figure 1.

### 3.2. Driver Proteins

Driver proteins are a subset of the network’s proteins that must be directly controlled to manipulate total system behavior. This subset, size $N_{D}$, is identified through maximum matching algorithms [18] and serves as the first step in both methods of controllability. Calculations identified $N_{D}$ = 2463 in the HIN and $N_{D}$ = 2466 in the VIN, implying that there is little change to the control structure of the network during infection. All SARS-CoV-2 proteins are driver proteins of the VIN. The 20 host proteins displaced by SARS-CoV-2 proteins as drivers are deemed “displaced proteins”. Their identities are listed in Table 1. Only five displaced proteins are not virus interacting proteins (HPR, CNNM3, TRIM51, DIP2A, MICA). The removal of these five proteins as drivers of infected cell behavior in the VIN suggests that they have fallen under the control of viral proteins or are part of a host cascade that has been activated in the response. Two proteins are of note: first, TRIM51 is a member of the tripartite interaction motif family of innate immunity regulators [20]. It is previously shown to be highly upregulated in the presence of TLR3 and TLR4 ligands [21] of the viral RNA sensing pathway [22]. Second, MICA is an MHC class I cell surface protein which regulates the activation of both T cells and natural killer cells during a stress response along with other NKG2D ligands such as RAE1 [23,24]. The displaced protein set was analyzed with Interferome v2.01 [25] to determine their status as interferon regulated genes (IRGs) known to exhibit a fold change in expression greater than two in interferon knockdown studies. All displaced proteins are IRGs with the exception of SIGMAR1, EIF42E, and MICA. The altered role of these immune proteins as drivers of network behavior is representative of the activation of immune response pathways.

### 3.3. Robust Controllability

A robust controllability analysis was performed on the HIN and VIN as described in Section 2 to determine the effect of singular protein components on total system behavior. The classification results are shown in Table 2. Aside from the addition of viral nodes, there is very little change to the robust controllability of the VIN as compared to the HIN. The majority of all proteins are classified as neutral (VIN: 42.4%, HIN: 42.3%) and dispensable (VIN: 39.0%, HIN: 39.1%), suggesting that most proteins are regulated by neighboring protein pathways (neutral) or make the network easier to control in their absence (dispensable). Conversely, the loss of a small proportion of indispensable proteins (VIN and HIN: 18.6%) would make the network increasingly difficult to regulate. The driver protein population is skewed toward those with dispensable classifications (VIN: 67.2%, HIN: 67.6%) as compared to all proteins. Classifications of virus interacting proteins are similar to those of the total network, eliminating the possibility that viral interactions target proteins that are advantageous to robust controllability. Viral proteins E, nsp5, and nsp10 are classified as dispensable in the VIN. All other viral proteins are classified as neutral.

The nine host proteins that change robust classification after the addition of viral interactions are listed in Table 3. Four of the “robust proteins” are also virus interacting proteins (IMPDH2, RAE1, SIGMAR1, NUP210) and three belong to the displaced protein set (IMPDH2, SIGMAR1, NUP210). While only the four virus interacting proteins exhibit an increase in degree from a singular viral interaction, all nine robust proteins demonstrate an increase in betweenness in the infected network, some by orders of magnitude. Of note, the betweenness of IMPDH2 increases from 65 to 4090 and SIGMAR1 reaches 4094 where it has a betweenness of 0 in the HIN. This trend demonstrates the importance of the robust protein set to network information flow and regulation in the infected cell. There is no trend in the changes to classification type for the robust group. Only two robust proteins were identified as IRGs with a fold change greater than two by Interferome (IMPDH2, DYNLT1).

**Figure 2:**
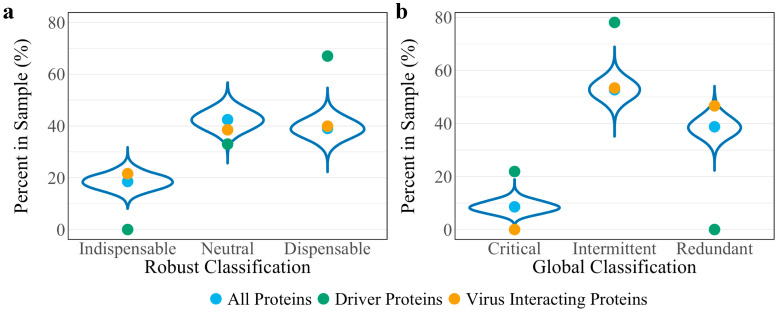
Distributions of controllability classification results of 10,000 random “pseudo-virus interacting” protein sets for (**a**) robust controllability and (**b**) global controllability. True values for all proteins (blue), driver proteins (green), and virus interacting proteins (yellow) of the VIN are shown as reference for each classification.

**Figure 3:**
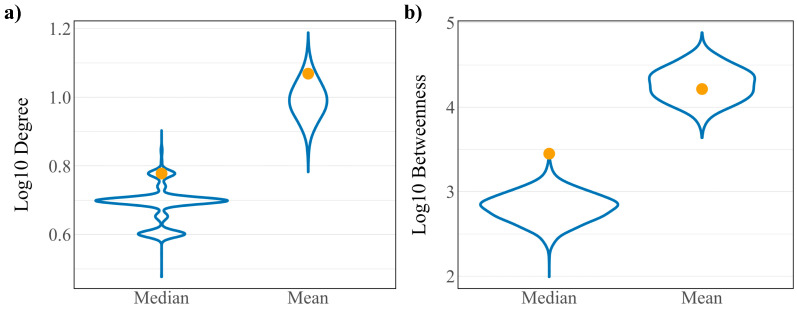
Distributions of (**a**) log_10_ degree and (**b**) log_10_ betweenness of 10,000 random “pseudo-virus interacting” protein sets. True values for virus interacting proteins are shown in yellow.

To assess whether the robust controllability classifications of the driver and virus interacting proteins are a result of the network’s connectivity structure, a randomization analysis was performed as developed in previous work [11]. A random set of 148 host proteins representing a “pseudo-virus interacting” protein set was pulled from the network and assessed for robust controllability. The resulting distributions from 10,000 iterations of this process are reported in Figure 2a against the true values for all proteins, driver proteins, and virus interacting proteins. Distributions are reflective of the true values of all proteins for all three robust controllability classifications. True values for virus interacting proteins also fall within the distributions for robust classifications implying that there is no regulatory advantage for the particular set of host proteins interacting with SARS-CoV-2 within the robust controllability framework. However, true values for driver proteins fall outside the distributions generated by the pseudo-sets, implying that the groups are distinctly different in regulatory function. A topological analysis including the median and mean values of the same distributions against the true values for virus interacting proteins is shown in Figure 3. The mean log_10_ degree and betweenness is significantly higher than the corresponding distribution mean (one-sided *t*-test *p*-values: 2.2 × 10^−16^, 2.2 × 10^−16^) implying that the virus prefers to interact with proteins that hold significance to network structure.

### 3.4. Global Controllability

Similarly, a global controllability analysis was performed on the HIN and VIN as described in Section 2. Results are shown in Table 4. As in the robust controllability analysis, global controllability classifications of the VIN’s proteins are almost identical to those of the HIN. Over half (VIN: 52.7%, HIN: 52.8%) of all proteins are classified as intermittent, suggesting that the majority of proteins are able to play a role in cellular regulation. Only a small percentage (VIN: 8.6%, HIN: 8.4%) of all proteins are classified as critical, meaning they are involved in all combinations of network regulators. By definition, driver nodes cannot be redundant, therefore, they are predominately classified as intermittent (VIN: 78.1%, HIN: 80.5%). Unlike the robust analysis, classifications of virus interacting proteins differ slightly from those of the total protein population. The eight critical virus interacting proteins of the HIN become intermittent in the VIN, losing some control over infected network regulation. There is a higher proportion of redundant virus interacting proteins in the VIN (46.6%) compared to both the virus interacting proteins in the HIN and all proteins of both the VIN and HIN (HIN virus interacting proteins: 32.9%, VIN all proteins: 38.7%, HIN all proteins: 38.8%), suggesting that proteins that directly interact with the virus are transitioning into deferential roles after the onset of infection. All 23 viral proteins are classified as critical in the VIN, always holding control of network regulation.

Eleven host proteins change classification after the addition of viral interactions (Table 5). All eleven “global proteins” interact with SARS-CoV-2 proteins with six belonging to the displaced protein set (SCARB1, IMPDH2, PVR, EIF4E2, SIGMAR1, and NUP210). Four global proteins are also identified as robust proteins (IMPDH2, RAE1, SIGMAR1, and NUP210). With the exception of EIF4E2, SIGMAR1, and SAAL1, all members of the set were identified as IRGs with a fold change greater than two by Interferome.

**Figure 4:**
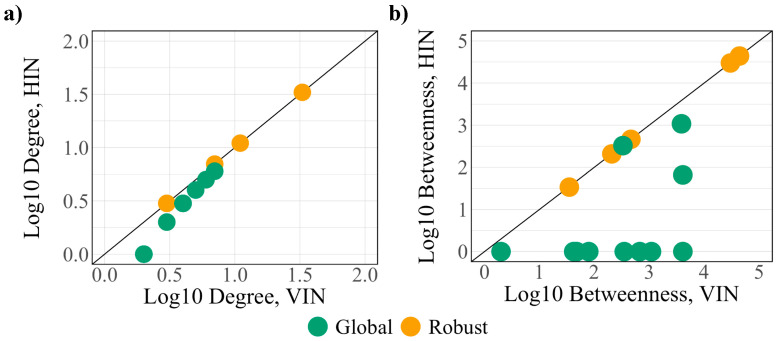
A comparison of the (**a**) degree and (**b**) betweenness of the robust (yellow) and global (green) protein sets.

The betweenness of the global protein set increases after the addition of virus–host interactions. In particular, the betweenness of eight of the global proteins (SCARB1, PVR, EIF4E2, CEP135, SIGMAR1, TOR1AIP1, RAB14, and SAAL1) is 0 in the HIN before increasing by several orders of magnitude in the VIN, implying that these proteins are integrated into the network information flow at the onset of infection. Supporting this, seven of the eight shift from critical to intermittent classification (SIGMAR1 becomes redundant) after the integration of viral interactions, indicating new regulation and a loss of control over the network. A topological comparison of the robust and global protein sets within the HIN and VIN (Figure 4) demonstrates larger differences between the degree and betweenness of the two networks, making the global controllability analysis a better predictor of the regulators of the infected cell.

A randomization analysis was performed as developed in the previous work [11] using the same “pseudo-virus interacting” protein sets assessed for robust controllability classifications. The resulting distributions from the 10,000 iterations are found in Figure 2b against the true values for all proteins, driver proteins, and virus interacting proteins. Again, random distributions are reflective of the true values from the global controllability of all proteins. While the true value for intermittent virus interacting proteins reflects the random distributions, true values for critical and redundant proteins fall at the tails of the distributions suggesting a regulatory advantage in SARS-CoV-2 interacting with redundant host proteins. True values for driver proteins fall outside the distributions generated by the pseudo-sets, supporting the conclusion that the groups are distinct.

### 3.5. Controllability-Guided Drug Targeting and Repurposing

Assuming the identified robust and global proteins are acting as regulators of the infected state, it follows that they have potential as drug targets for SARS-CoV-2 infection treatments. The Drugbank database [26] was used to prioritize the predicted proteins for drug repositioning efforts by assessing which proteins act as targets for existing drugs. Results were compared with the results of drug repurposing and viral inhibition studies performed by Gordon et al. [7]. Of the 16 combined robust and global proteins, six are drug targets registered in Drugbank (PVR, SCARB1, NUP210, SIGMAR1, IMPDH2, and EIF4E2). NUP210, SIGMAR1, IMPDH2, and EIF4E2 were identified by Gordon et al., though Drugbank identified compounds for each target that were not included in the viral inhibition studies. The compounds associated with two additional targets that were identified by the controllability methods but are unregistered in Drugbank (LARP1 and RAE1) were also previously identified. The targets and associated compounds for all eight genes are found in Supplementary Table S1.

A summary of compounds known to target PVR and SCARB1, i.e., the prioritized proteins that have not been recommended in previous repurposing studies are found in Table 6. PVR is a known regulator of natural killer cell adhesion to host cells and lytic granule secretion after binding to DNAM-1, a receptor expressed by natural killer cells, T cells, and monocytes [27]. First identified in the context of polio virus, it has also been identified for its role in motility during tumor cell invasion [28]. It is a member of the displaced driver set and the global set and acts as the target for two experimental compounds: myristic acid and sphingosine. A previous study of cytokine storms resulting from influenza virus infection asserts that the use of sphingosine-1-phosphate successfully blunts the overactive inflammatory response, limiting morbidity and mortality [29]. Given the similarly aggressive inflammatory response seen clinically in SARS-CoV-2 infected individuals [30], the prioritization of PVR is noteworthy.

SCARB1 is a cellular membrane protein involved in high-density lipid transport [31] that mediates cell entry of hepatitis C virus as the receptor for the E2 protein [33]. It was identified as both a displaced driver and global protein, and functions as the target for three compounds: phosphatidylserine, tocopherol/vitamin E, and PHA-665752. While not specific to viral infection, SCARB1 acts as a phosphatidylserine receptor on testicular Sertoli cells which induce phagocytosis of spermatogenic cells [34]. SCARB1 also acts as one of the most important transport vehicles for vitamin E in the lung’s alveolar cells, the presence of which largely regulates the receptor’s expression [35]. Vitamin E is also known to have a positive effect on influenza A viral clearance in the lungs of mice [36].

In addition to SCARB1, PHA-665752 targets NUP210, a nucleopore protein identified in all three protein sets of interest from controllability. Knockout experiments reveal that NUP210 has wide effects on T cell differentiation and response [32]. While not studied in the context of viral infection, PHA-665751 is known to induce apoptosis in both tumor and vascular endothelial cells resulting from non-small cell lung cancer [37].

SIGMAR1 and IMPDH2 were also identified in all controllability predicted groups. Sigma receptors 1 and 2 (SIGMAR1 and SIGMAR2) have been discussed as regulators of cellular stress and the apoptotic response [38]. Several known targeting compounds show evidence of inhibition at the viral replication stage including haloperidol, PB28, and widely discussed hydroxychloroquine. Many Drugbank predicted compounds targeting SIGMAR1 have not been tested for viral inhibition. IMPDH2 has long been a goal for targeting with immunosuppressive treatments, though inhibition with small molecules is notoriously difficult [39]. While many Drugbank-predicted compounds for IMPDH2 have not been tested in this context, mycophenolic acid and ribavirin were assessed in the viral inhibition screen with only the latter displaying active inhibition.

EIF4AE belongs to the 4E family of translation initiation factor proteins which bind to mRNA 5’ cap structures to recruit ribosomal recruitment within the cytosol [40]. The 4E family controls the rate of the early steps of the protein translation process. EIF4AE is a member of both the displaced driver set and the global protein set. A different translation regulator, LARP1, was identified as a druggable target for SARS-CoV-2 by Gordon et al., given previous evidence that the downstream effects of a common kinase inhibitor, rapamycin, inhibited MERS-CoV infection by over 60% [41]. Studies show that rapamycin promotes the phosphorylation of EIF4AE, achieving similar inhibition of the mTOR pathway [42]; however, evidence of viral inhibition after rapamycin treatment was inconclusive. Still, several other mRNA translation inhibitor compounds such as ternatin 4 and zotatifin have tested as active inhibitors of SARS-CoV-2, making EIF4AE an interesting prospect for future study.

## 4. Discussion

Here, a set of drug targets is prioritized for drug repurposing efforts in the global fight against COVID-19. Network controllability methods, with only disease-specific virus–host and host–host protein interaction data, create a large-scale representation of regulatory changes occuring during infection. With no additional biological information, the connectivity of the network is sufficient to predict the most biologically relevant components of the disease system, as evidenced by the high level of overlap between the presented results and the extensive biological analysis performed in Gordon et al.’s study for SARS-CoV-2 [7]. In total, this study demonstrates a simple computational approach to prioritizing drug target predictions with minimal biological context, an advantage in present times where viral understanding and data is even more sparse than usual.

As seen in the previous study of influenza A virus [11], the magnitude of control needed to manipulate the total cell system (number of driver proteins) is comparable in the healthy and infected cellular networks. The small changes in driver proteins between the networks are seen in immunoregulatory proteins that are typically upregulated during viral infection (such as TRIM51 and MICA) and many of the proteins identified in the controllability analyses. This is reflective of the activation of the immune response pathways and their effect on the cell as a whole.

With respect to the ratio of resultant classifications in both controllability methods, outcomes are again similar to those achieved with the influenza A virus–host network [11]. This is unsurprising due to the use of the same host network in the analyses. However, the low overlap between the controllability predicted proteins for the two diseases (3/16 proteins, PVR, RAB14, and SAAL1) demonstrates that while the method is easily applied to other viruses, the result is unique.

One limitation of this method is the requirement of high-confidence virus–host protein interaction data where the host proteins exist in the HIN. As experimentally-validated, directed networks are typically smaller than the available undirected networks, the method was unfortunately unable to use over half of the 332 known SARS-CoV-2–host protein interactions (in comparison, the influenza A virus network contains 752 virus–host interactions). Even so, the controllability analysis was able to predict biologically relevant proteins involved in functions like the cellular stress response, host translation, and cellular transport, proving the robustness of the method.

Of the eight prioritized targets, all but NUP210 exhibit large increases in betweenness after the addition of virus–host protein interactions, placing particular importance on their role in infected cell behavior based on topology. Further, most of the global protein set (including the novel predictions, PVR, and SCARB1) have a betweenness of zero in the HIN, implying that their individual significance to cellular network flow is truly unique to the infected cell. With the majority of the identified proteins being regulated by the interferon response (with a fold change in expression greater than two), this network result translates to immunological significance. The alterations in classification for all global proteins indicate a step down in network control where critical proteins have the most control and redundant the least. Biologically, this could represent viral interruption of normal host function or activation of a new pathway, both being interesting prospects for drug development.

Given the biological relevance of the topologically predicted/controllability target proteins, there is a good reason to pursue these recommendations, either in drug repurposing or in novel drug development. The extended list of untested compounds found in Supplementary Table S1 will be considered for further viral inhibition studies, particularly tocopherol/vitamin E which is already approved and has documented positive effects on viral clearance for influenza A [36]. Predictions indicate opportunity to both interfere with the viral replication cycle or to modulate the immune response to infection. Therefore, to most efficiently translate these findings to bedside, knockdown studies or siRNA screens should be used to validate drug predictions for each target. Cell culture studies that track interferon and cytokine activity may further establish a possible mechanism between the proposed targets and immune regulation. By narrowing the pool of drug target candidates with controllability methods, experimental validation will be efficient and timely.

## Figures and Tables

**Table 1:** The identities of the displaced driver proteins: the proteins that are drivers in the HIN, not the VIN. If the proteins are also virus interacting, viral protein interactor is given along with status as an interferon regulated gene (IRG).

Entrez ID	Gene Name	Virus Interaction	IRG
3250	haptoglobin-related protein (HPR)	-	X
23225	nucleoporin 210 (NUP210)	Nsp4	X
26505	cyclin and CBS domain divalent metal cation transport mediator 3 (CNNM3)	-	X
5557	primase (DNA) subunit 1 (PRIM1)	Nsp1	X
23367	La ribonucleoprotein domain family member 1 (LARP1)	N	X
382	ADP ribosylation factor 6 (ARF6)	Nsp15	X
2802	golgin A3 (GOLGA3)	Nsp13	X
949	scavenger receptor class B member 1 (SCARB1)	Nsp7	X
10280	sigma non-opioid intracellular receptor 1 (SIGMAR1)	Nsp6	
84767	tripartite motif-containing 51 (TRIM51)	-	X
3615	inosine monophosphate dehydrogenase 2 (IMPDH2)	Nsp14	X
9470	eukaryotic translation initiation factor 4E family member 2 (EIF4E2)	Nsp2	
55823	VPS11, CORVET/HOPS core subunit (VPS11)	ORF3a, ORF8	X
523	ATPase H+ transporting V1 subunit A (ATP6V1A)	M	X
2876	glutathione peroxidase 1 (GPX1)	Nsp5_C145A	X
23181	disco interacting protein 2 homolog A (DIP2A)	-	X
2150	F2R like trypsin receptor 1 (F2RL1)	ORF9c	X
5817	poliovirus receptor (PVR)	ORF8	X
6731	signal recognition particle 72 (SRP72)	Nsp8	X
4276	MHC class I polypeptide-related sequence A (MICA)	-	

**Table 2:** The number of proteins in each robust controllability category for all proteins, driver proteins, and SARS-CoV-2 interacting proteins. Values are reported as totals and percent total for the VIN (HIN).

	Indispensable	Neutral	Dispensable	Total
All proteins	1170 (1169)	2675 (2658)	2459 (2454)	6304 (6281)
	18.6% (18.6%)	42.4% (42.3%)	39.0% (39.1%)	100%
Driver proteins	0 (0)	810 (799)	1656 (1664)	2466 (2463)
	0% (0%)	32.8% (32.4%)	67.2% (67.6%)	100%
Virus interacting proteins	32 (30)	57 (57)	59 (61)	148 (148)
	21.6% (20.3%)	38.5% (38.5%)	39.8% (41.2%)	100%

**Table 3:** The identities and topological characteristics of the proteins identified in the robust controllability analysis. Values for degree, betweenness, and classification are given as VIN (HIN). Classification is denoted as indispensable, I; neutral, N; and dispensable, D. Genes which have experimentally shown a fold change in expression greater than 2 during interferon knockdown studies are denoted as interferon regulated genes (IRGs).

Entrez ID	Gene Name	Degree	Betweenness	Classification	IRG
1174	adaptor-related protein complex 1 sigma 1 subunit (AP1S1)	3 (3)	34.1 (33.0)	D (N)	
3615	inosine monophosphate dehydrogenase 2(IMPDH2)	4 (3)	4090.0 (65.0)	N (D)	X
6993	dynein light chain Tctex-type 1(DYNLT1)	11 (11)	44,093.7 (43,902.7)	D (N)	X
8480	ribonucleic acid export 1(RAE1)	6 (5)	3863.3 (1076.3)	I (N)	
10280	sigma non-opioid intracellular receptor 1(SIGMAR1)	3 (2)	4094.0 (0.0)	I (N)	
10987	COP9 signalosome subunit 5(COPS5)	33 (33)	30,094.4 (29,968.0)	N (I)	
23225	nucleoporin 210(NUP210)	4 (3)	331.1 (325.9)	N (D)	
64326	ring finger and WD repeat domain 2(RFWD2)	3 (3)	207.1 (206.8)	D (N)	
64837	kinesin light chain 2(KLC2)	7 (7)	463.2 (460.3)	D (N)	

**Table 4:** The number of proteins of each global controllability classification for all proteins, driver proteins, and SARS-CoV-2 interacting proteins. Values are reported as totals and percent total for the VIN (HIN).

	Critical	Intermittent	Redundant	Total
All proteins	540 (525)	3322 (3318)	2442 (2438)	6304 (6281)
	8.6% (8.4%)	52.7% (52.8%)	38.7% (38.8%)	100%
Driver proteins	540 (525)	1926 (1983)	0 (0)	2466 (2463)
	21.9% (21.3%)	78.1% (80.5%)	0% (0%)	100%
Virus interacting proteins	0 (8)	79 (75)	69 (65)	148 (148)
	0% (5.4%)	53.4% (50.7%)	46.6% (32.9%)	100%

**Table 5:** The identities and topological characteristics of the proteins identified in the global controllability analysis. Values for degree, betweenness, and classification are given as VIN (HIN). Classification is denoted as indispensable, I; neutral, N; and dispensable, D. Genes which have experimentally shown a fold change in expression greater than 2 during interferon knockdown studies are denoted as interferon regulated genes (IRGs).

Entrez ID	Gene Name	Degree	Betweenness	Classification	IRG
949	scavenger receptor class B member 1(SCARB1)	5 (4)	1098.0 (0.0)	I (C)	X
3615	inosine monophosphate dehydrogenase 2(IMPDH2)	4 (3)	4090.0 (65.0)	R (I)	X
5817	poliovirus receptor (PVR)	7 (6)	349.6 (0.0)	I (C)	X
8480	ribonucleic acid export 1(RAE1)	6 (5)	3863.3 (1076.3)	R (I)	X
9470	eukaryotic translation initiation factor 4E family member 2(EIF4E2)	4 (3)	672.1 (0.0)	I (C)	
9662	centrosomal protein 135(CEP135)	2 (1)	46.6 (0.0)	I (C)	X
10280	sigma non-opioid intracellular receptor 1(SIGMAR1)	3 (2)	4094.0 (0.0)	R (C)	
23225	nucleoporin 210(NUP210)	4 (3)	331.1 (325.9)	R (I)	X
26092	torsin 1A interacting protein 1(TOR1AIP1)	2 (1)	41.1 (0.0)	I (C)	X
51552	RAB14, member RAS oncogene family (RAB14)	2 (1)	1.0 (0.0)	I (C)	X
113174	serum amyloid A like 1(SAAL1)	2 (1)	78.2 (0.0)	I (C)	

**Table 6:** Status and structure of drugs known to target controllability predicted proteins. Each target’s SARS-CoV-2 protein interactor is given along with its known target function.

Drug (Status)	Structure	Target/Viral Protein	Target Function
Myristic acid (Experimental)	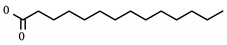	PVR/ORF8	Regulate Natural killer cells, polio virus [27]
Sphingosine (Experimental)	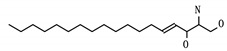		
Phosphatidyl serine (Approved)	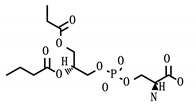	SCARB1/Nsp7	Facilitate cell entry, Hepatitis C [31]
Tocopherol/Vitamin E (Approved)	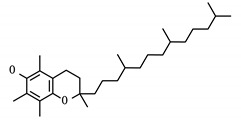		
PHA-665752 (Experimental)	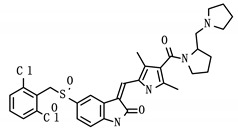	SCARB1/Nsp7	Facilitate cell entry, Hepatitis C [31]
		NUP210/Nsp4	Transport between nucleus and cytoplasm [32]

## Supplementary Materials

The following are available online at https://www.mdpi.com/1999-4915/12/10/1087/s1, Table S1: Compounds targeting controllability-identified targets.
